# The lived experience of mothers who give birth after induction of labour in hospital settings in Southern Ethiopia: a phenomenological study

**DOI:** 10.1186/s12978-025-02054-6

**Published:** 2025-07-10

**Authors:** Adanech Abebe, Temesgen Nane Setato, Fithamlak Solomon Bisetegn, Samson Kastro Dake, Debritu Nane

**Affiliations:** 1 College of Health Science and Medicine, School of Public Health, Department of Reproductive Health and Nutrition, Wolaita Sodo University, Wolaita Sodo, Ethiopia; 2 College of Health Science and Medicine, Department of Surgery, Pediatric Surgery Division, Wolaita Sodo University, Sodo, Ethiopia; 3 School of Medical Laboratory, Wolaita Sodo University, Wolaita Sodo, Ethiopia

**Keywords:** Phenomenology, Lived experience, Mothers, Induction of labor

## Abstract

**Background:**

Induction of labor (IOL) is a medical intervention used to initiate labor artificially and has become one of the most common procedures in modern obstetrics to improve maternal and neonatal outcomes. While extensive research has focused on the methods and outcomes of labor induction, limited evidence exists regarding mothers’ lived experiences following IOL, particularly in the Ethiopian context.

**Objective:**

To explore the lived experience of mothers who give birth after induction of labor at Wolaita Sodo Comprehensive Specialized Hospital, Southern Ethiopia, 2024.

**Methods:**

A qualitative descriptive phenomenological study was conducted at Wolaita Sodo Comprehensive and Specialized Hospital between September and November 2023. Twelve postpartum mothers were purposively selected for in-depth interviews using a semi-structured guide. Interviews were audio-recorded, transcribed verbatim, translated into English, and supplemented with field notes. Data were analyzed thematically using Braun and Clarke’s six-phase method, supported by Open Code Software Version 4.02. Trustworthiness was ensured through the application of Guba’s criteria: credibility, transferability, dependability, and confirmability.

**Result:**

Four main themes emerged: (1) emotional responses to IOL, (2) coping with IOL and support received, (3) challenges encountered during the induction process, and (4) perceived effects of IOL on the mother. Participants reported intense negative emotions such as pain, fear, anxiety, and distress. These feelings were exacerbated by systemic challenges, including overcrowded wards, lack of privacy, and inadequate environmental conditions in the labor ward.

**Conclusion and recommendation:**

Mothers undergoing labor induction experience both positive and negative emotions. The stressful environment of the labor ward and the induction process negatively affect their emotional well-being. To improve maternal care outcomes, it is essential to foster effective communication between healthcare providers and mothers and to address the environmental challenges within the hospital setting.

## Introduction

Maternal and child health remains a major global public health concern. Induction of labor (IOL), defined as the artificial initiation of uterine contractions before the spontaneous onset of labor, is a common obstetric intervention used to reduce maternal and neonatal morbidity and mortality in the presence of complications such as post-term pregnancy, hypertensive disorders, or premature rupture of membranes (PROM) [1].

According to the World Health Organization (WHO), approximately 25% of term deliveries in developed countries involve IOL, whereas the average rate in developing countries is considerably lower, at about 4.4% (Lidet L: Prevalence and factor associated with successful induction of labor among mothers delivered in Debre Berhan comprehensive specialized hospital, 2020 GC a retrospective cross sectional study, unpublished). The WHO recommends that labor induction should be primarily indicated to enhance the quality of care and outcomes for pregnant women undergoing the procedure, especially in resource-limited settings [2].

Indications for labor induction included gestational hypertension, pre-eclampsia/eclampsia, gestational diabetes mellitus (GDM), PROM, post-term pregnancy, fetal demise, maternal medical conditions such as autoimmune diseases, and fetal complications like small for gestational age and fetal anomalies [3, 4]. The primary indications are post-term pregnancy, increased risk of stillbirth, microsomia, birth injury, meconium aspiration syndrome, PROM, and hypertensive disorders [5].

While IOL has proven benefits, such as reducing the risks of stillbirth and infection, it is also associated with complications. These include induction failure, uterine hyper-stimulation, increased cesarean delivery rates, postpartum hemorrhage (PPH), and adverse neonatal outcomes [4, 6].

Women undergoing IOL are less likely to be satisfied with their care and childbirth experience [7] and more often express fear, anxiety, dissatisfaction with care, and feelings of neglect [6]. These psychological responses, combined with physical exhaustion, lack of sleep, and discomfort from the procedure, may adversely affect maternal well-being during labor and the postpartum period [5, 8].

IOL when compared to natural vaginal labor increase complications such as longer hospital stays, a more need for anti pain, a greater risk of infection and problems, as well as poor maternal and perinatal outcomes [9]. Studies conducted in Germany and Australia report that adverse effects of labor induction include excessively rapid and painful labor, frustrating induction failure, increased incidence of meconium-stained amniotic fluid, uterine rupture, uterine hyper-stimulation, amniotic fluid embolism, maternal death, perinatal mortality, hypoxic-ischemic injury, and severe birth asphyxia [6, 10].

Despite the widespread use of IOL in Ethiopia as part of national efforts to reduce maternal mortality and achieve Sustainable Development (SDG) Goal 3.1, there is limited qualitative evidence on how mothers emotionally and psychologically experience the process of IOL. Most existing studies focus on clinical outcomes, overlooking the subjective perspectives of the women themselves. Therefore, this study aims to explore the lived experiences of mothers who underwent induction of labor at Wolaita Sodo University Comprehensive Specialized Hospital, Southern Ethiopia. Understanding these experiences is critical to improving maternal care and supporting women during IOL.

## Materials and methods

### Study setting

This study was conducted at Wolaita Sodo University Comprehensive and Specialized Hospital (WSUCSH) in Wolaita Zone, located 330 km southwest of Addis Ababa, the capital city of Ethiopia. Wolaita Zone is projected to have more than 2.6 million people in 2020 [11]. The hospital serves approximately 2–6 million people from Wolaita and surrounding areas. The obstetric department includes 15 units, one of which is the labor ward, staffed by 10 midwives, 10 gynecologists, resident doctors, and intern medical students. The ward has 11 beds and, over a six-month period, approximately 1,737 deliveries occurred, with 238 resulting from IOL.

### Study design and period

A qualitative descriptive phenomenological study was conducted from September to November 2023 to explore the lived experiences of mothers who gave birth following IOL.

### Sampling and participants

Participants included mothers who gave birth to healthy neonates via IOL during the study period. Mothers experiencing obstetric complications such as fetal loss, preterm birth, or fetal anomalies were excluded. Homogeneous purposive sampling was employed, and recruitment continued until data saturation was achieved. Eligible participants were identified by ward charge midwives and referred to the research team. Those who agreed to participate received detailed study information and provided informed consent.

### Data collection process

A pilot test involving two eligible participants was conducted to refine the interview guide [12]. Data were collected through face-to-face, in-depth interviews using a semi-structured interview guide. Interviews were conducted in private rooms within the obstetric ward and were audio-recorded with participants’ consent. Interviews were held in Amharic or Wolaitato, depending on the participant’s preference, and then back-translated into English to ensure accuracy.

The principal investigator (PI) conducted the interviews, with assistance from a trained researcher who managed audio recording and note-taking. Probing questions were used to elicit detailed accounts. Interviews lasted 45–60 min and continued until thematic saturation was achieved (*n* = 12). Field notes were also taken to capture nonverbal cues and contextual details.

### Trustworthiness

To ensure the trustworthiness of the study, the criteria of credibility, transferability, dependability, and confirmability were carefully maintained. Credibility was supported through prolonged engagement with participants and peer debriefing was also conducted with a colleague in a similar professional role to validate the findings and enhance the study’s rigor. Transferability was enhanced by using purposive sampling and providing thick descriptions of the study context. Dependability was ensured by maintaining consistency in data transcription and storage, and triangulation of data sources (verbal and nonverbal). Confirmability was achieved by adhering to systematic procedures during data collection, analysis, and reporting. An audit trail was maintained, and direct quotations from participants were included to support and validate the findings. Senior supervisors also reviewed the audit trail to enhance objectivity.

To support bracketing, a literature review was conducted only after data collection and analysis to prevent prior knowledge from influencing the participants’ responses. Additionally, a reflexive diary was kept throughout the study to document the researchers’ assumptions, thoughts, and potential biases, helping to maintain neutrality and focus on the participants’ lived experiences.

### Data analysis

Data collection and analysis were conducted concurrently using Braun and Clarke’s six-phase thematic analysis framework to ensure a rigorous and systematic process [13].

In the first phase, researchers familiarized themselves with the data by reviewing audio recordings immediately after each interview, transcribing them verbatim, and translating the transcripts into English. This was followed by a thorough reading of the translated transcripts to internalize participants’ experiences and begin noting initial ideas. In the second phase, preliminary analysis was conducted to assess data saturation and develop a code-book that guided the coding process. In the third phase, translated transcripts were uploaded into Open Code software version 4.02, and initial codes were generated systematically. Three assistant researchers with prior experience in qualitative research independently coded the same transcripts using the predefined code-book. To ensure inter-coder reliability, their coding results were compared and discussed. Discrepancies were resolved through consensus during regular debriefing sessions with the principal investigator, and the code-book was refined accordingly.

In the fourth phase, themes were reviewed collaboratively to ensure consistency and coherence across the datasets. This process helped refine the thematic structure and validate that the themes captured the essence of participants’ narratives. In the fifth phase, the themes and sub-themes were clearly defined and named to reflect their underlying meanings. Finally, in the sixth phase, the findings were organized into major themes and sub-themes and presented with direct quotations from participants to illustrate key concepts. Thick descriptions of the study context were provided to support the interpretation of findings, and member checking was conducted by reviewing the transcripts with the participating women to confirm the accuracy of interpretations and enhance the credibility of the analysis.

## Result

### Socio-demographic characteristics of the participants

Twelve married women participated in the study, aged between 22 and 35 years. Most lived in urban areas, with only one residing in a rural setting. Educational levels varied: five had first degrees, two held diplomas, three completed secondary school, and two had primary-level education. Half of the participants were housewives, while others worked in government or private sectors. Monthly income ranged from 3,000 to 18,000 Ethiopian birr (ETB). Most women were Protestant Christians, with a few identifying as Orthodox. The number of children ranged from one to five. Detailed socio-demographic characteristics are presented in (Table 2 in Appendix).

### Identified themes

In this study, 92 codes, 4 themes, and 8 sub-themes were identified concerning the lived experiences of mothers who give birth after IOL. The extracted themes were: emotional responses to IOL, coping with IOL and support received, challenges encountered during the induction process, and perceived effects of IOL on the mother (Table 1).

**Table 1:** Emergent themes and sub-themes from participant interviews, WSUCSH, Southern Ethiopia, 2024

Themes	Sub-themes
Emotional responses to IOL	✓ Feelings of pain, fear, anxiety, and worry ✓ Feeling happiness after gave birth successful
Coping with IOL and support received	✓ Care and help from doctors and midwives ✓ Family care and help
Challenges encountered during the induction process	✓ The stressful labour ward environment ✓ Stressful process of induction
Perceived effects of IOL on the mother	✓ Effect on the physical body ✓ Effect on the psychological mind

### Theme 1: emotional responses to IOL

Mothers shared a spectrum of emotional experiences ranging from fear and anxiety to relief and happiness. Most were unprepared for the emotional toll of IOL. The emotional experiences of mothers are centered around two key sub-themes: the feelings of fear, anxiety, pain, and worry, and the sense of happiness following a successful birth.

#### Sub-theme: feelings of pain, fear, anxiety, and worry

The anticipation and initiation of induction caused significant distress. Common fears included the pain of induction, concern for the baby’s health, and uncertainty due to misinformation. In this study, almost all women reported that they were worried and feared when they heard about the IOL and starting it. They were rarely prepared for the emotional stresses. Mother’s shared that;

*“I became anxious when they told me that labor needed to be induced… I was deeply shocked by the situation.”* (Participant 2, 27 years).

*“Labor pain from induction is very different from normal delivery pain; it is extremely severe. When I gave birth to my first child through normal labor*,* the pain was so intense that I don’t even want to remember it. I was in so much pain that I lost track of where I was*,* whether I was in bed or somewhere else. I was terrified I might die; it felt like something was squeezing me tightly*,* moving me up and down. It seemed like I needed to urinate but couldn’t*,* even though they kept telling me the baby was coming soon. I prayed for help.”* (Participant 1, 25 years old mother).

These experiences reflect the heightened emotional and psychological burden induced labor brings, often worsened by misinformation and lack of preparation.

#### Sub-theme: feeling happiness after successful birth

Despite initial fear and discomfort, the successful outcome brought joy and a sense of relief.

*“I am not so tired*,* and the child born is safe… I am so happy and bless GOD for this good outcome.”* (Participant 4, 29 years old mother).

*I am delighted because I was concerned about my fetus’s health… I gave birth vaginally*,* and everything went well.”*(Participant 5, 30 years old mother).

This emotional shift highlights how positive outcomes can alleviate the distress associated with induction of labor, providing a sense of closure and fostering feelings of gratitude.

### Theme 2: coping with IOL and support received

The care and help that mothers received is categorized into two sub-themes: care and help from the doctors and midwifes; and family care and help.

#### Sub-theme: care and help from the doctors and midwives

Most mothers reported receiving compassionate and attentive care from healthcare providers, which played a crucial role in easing their fear and distress.

*“There was fear that my son would die. But I am ok with the help of doctors and midwifes. They come to me and reassure me… monitoring the health of my child and me frequently. GOD bless them.”* (Participant 1, 25 years old mother).

*“The process is going well. The doctors are right here with me*,* closely monitoring everything. They are tracking everything meticulously*,* listening to the baby’s heartbeat*,* and keeping a close eye on all the details. From what they have told me*,* they are saying that everything is successful.”* (Participant 9, 27 years old mother).

Such support helped the mothers feel safe and cared for during the stressful labor experience.

#### Sub-theme: family help and care

Family members played a vital role in helping mothers manage the stressful period of labor induction. Their support greatly improved the mothers’ capacity to cope with the situation. Families made frequent visits, expressed their emotions, and provided comfort and encouragement, which helped mothers accept the doctors’ decisions and stay resilient.

Mothers expressed their families’ support as:

*“The doctor suggested that I hold onto the bed and consult with my husband. At first*,* I resisted*,* but my husband encouraged me to trust the doctor’s advice*,* reminding me that they understood the situation better. Eventually*,* I accepted his encouragement and followed the recommendation….”* (Participant 12, 35 years old mother).

*“..My mother stayed with me throughout. When I cried out in pain*,* she cried too*,* massaging my back constantly*,* and encouraging me to stay strong.”* …” (Participant 10, 35 years old mother).

### Theme 3: challenges encountered during the induction process

The primary difficulties identified as sub-themes were the stressful environment of the labor ward and the stressful nature of the induction process.

#### Sub-theme: stressful labour ward environment

During their stay in the labor ward, mothers reported that the environment was stressful. They expressed concerns about overcrowding, noise, lack of privacy, and the suffocating conditions of the room. Overcrowding was especially highlighted as a major concern. Mothers shared their labour ward environment experience as:

*“Since I was transferred to this room after delivery*,* I feel worried when the room is overcrowded by people*,* and after delivery*,* I feel worried when I hear the mothers screaming*,* and it was difficult to urinate*,* change clothes*,* and eat food.”* (Participant 5, 30-year-old mother).

*“The room is very noisy. It was a very disturbing noise*,* as there are different mothers with different causes and not only mothers having induction. The room is stressful*,* as I am also much stressed.”* (Participant 7, 29 years old mother).

#### Sub-theme: stressful process of induction

Most mothers reported that vaginal examinations, including catheter insertion and frequent fetal heartbeat monitoring with a fetoscope, were uncomfortable and extremely stressful experiences during IOL.

*“When they first inserted a tube into my uterus to soften and dilate it*, *it was painful and stressful. I experienced cramping when labor began*,* which was very stressful*,* and the pain was severe.”* (Participant 4, 27-year-old mother).

*“However*,* in the IOL*,* frequent examination by fetoscope is painful to the bladder; they frequently insert their finger into the vagina*, * and that is very difficult. Even medication has cramps*,* and there is fatigue after medication administration.”* (Participant 10, 27-year-old mother).

### Theme 4: perceived effects of IOL on the mother

The labour started with induction affected mothers during labour and postpartum time. IOL has a notable impact on mothers, influencing both their physical and psychological well-being during labor and the postpartum period. The experiences of mothers highlight two key aspects: the physical strain and the psychological stress associated with IOL.

#### Sub-theme: effect on the physical body

Many participants reported that the induced labor caused prolonged pain and physical exhaustion, sometimes interfering with postpartum recovery.

*“… when normal labour begins*,* the uterus quickly snaps back into place on its own. But labour started with an induction*,* from what I understand*,* the uterus may not return quickly on its own. Bleeding may not stop soon. Still*,* I have the pain. My whole body is trembling to move. I have a hard time getting up to urinate and going back to the place. My waist is very painful. It was hard to breastfeed the baby. I was very dizzy.”* (Participant 12, 35 years old mother).

*“After I delivered… I couldn’t pass urine or stool for six hours. My husband had to support me even to eat.”* (Participant 11, 26 years old mother).

Such statements highlight the debilitating physical effects that induction may cause, indicating a need for tailored postpartum care.

#### Sub-theme: psychological effect

Many mothers described how misinformation often obtained from peers or the community combined with the intensity of labor pain, significantly affected their emotional well-being, particularly in the initial hours. One participant recounted:

*“The reality was very different from what I had heard before. I had tried to give birth before*,* and it was extremely stressful*,* it made me feel like I was going crazy. They said I needed glucose because the stress overwhelmed me. People also told me it involved a lot of risk.”* (Participant 4, 27 years old mother).

*“Cramping*,* vomiting*,* headache… I was lost and even insulted others.”* (Participant 4, 27 years old mother).

## Discussion

In this study, we explored the lived experiences of mothers who give birth following IOL. Four main themes emerged from the narratives: emotional responses to IOL, coping with IOL and support received, challenges encountered during the induction process, and perceived effects of IOL on the mother.

Our findings reveal that mothers experienced a spectrum of intense emotions, ranging from pain, fear, anxiety, and worry to joy, happiness, and hope throughout their induction and childbirth journey. These emotional responses were often shaped by their prior knowledge, expectations, and the quality of interaction with healthcare providers. Such emotional variability aligns with previous studies from Australia [14], a systematic review of qualitative studies [15], and research from the UK [16, 17], suggesting that childbirth following IOL is a globally complex and emotionally charged experience.

Negative emotional responses were often rooted in the unexpected nature of induction, the pain associated with artificial initiation of labor, and fear of adverse outcomes. Similar to findings with research from the University of Malta [18], where participants used terms like “painful experience,” “it wasn’t successful,” and “very annoying” to describe their IOL experiences. This underscores the critical need for antenatal education about IOL, including realistic expectations and pain management strategies. As supported by literature [1], unfamiliarity with the procedure can heighten anxiety, reinforcing the role of counselling and psychological preparation.

Conversely, many mothers in our study described joy and relief upon seeing their newborns, which helped them reinterpret the preceding pain more positively. This finding mirrors research from Sweden [1], where the sight of a healthy baby helped mothers re-frame their experience. These outcomes highlight the importance of emotional closure and suggest that achieving a positive birth outcome can mitigate some of the distress related to induction. This also points to the critical role of continuous care and support from health professionals throughout the process to get the positive birth outcome.

Our findings indicate that labor induction affected mothers both physically and psychologically during labor and the postpartum period. Induction of labor impacted mothers’ physical and emotional well-being, significantly influencing their lived experiences. Mothers reported feeling physically exhausted and mentally stressed. This is supported by research from Germany, which found that mothers felt their bodies were pushed to labor and deliver, regardless of their readiness [19]. This suggests that induction affects postpartum life compared to spontaneous labor, highlighting the need for counseling about the postpartum effects of induction.

Our study also revealed that mothers appreciated the support from healthcare providers, who offered care during labor and continued to support them through the postpartum period. Providers offered crucial informational and emotional support, guidance on managing IOL, and monitored the health of both the mothers and their fetuses.

The support mothers received played a central role in their ability to cope with the induction and childbirth process. Many women valued the care and reassurance from healthcare providers, who offered emotional support, health monitoring, and guidance. This care fostered trust and helped mothers navigate the stress of labor. Our findings are consistent with studies from Norway and Ghana, which highlighted the positive impact of compassionate and woman-centered care in improving the IOL experience [1, 15]. Continuous support, particularly from midwives, has been shown to improve maternal outcomes and satisfaction with the birth process [20].

However, while professional care was appreciated, mothers’ individual coping mechanisms were less often discussed in the literature but emerged as important in our study. Some women relied on faith, internal reassurance, or support from family members, where permitted. Unfortunately, the lack of companionship due to overcrowding, limited mothers’ ability to rely on familiar emotional supports, which has been noted as a major gap in facility-based care across low-resource settings [21].

The challenges mothers encountered during IOL were multifaceted. Environmental stressors such as overcrowded labor wards, noise, lack of privacy, and stuffy conditions were commonly reported and perceived as distressing. These findings are supported by research from Ghana, Sweden, and Denmark, which similarly noted that physical environments contribute significantly to maternal stress during labor [6, 22]. Addressing such infrastructural and systemic issues could substantially improve the maternal experience.

Additionally, routine procedures such as catheter insertions, and continuous fetal monitoring were often described as uncomfortable as painful or uncomfortable. These procedures were described as stressful and unpleasant. This resonates with studies from Sweden and Ghana, where mothers identified frequent vaginal exams and fetal monitoring as traumatic were the most painful and caused significant discomfort and psychological trauma [1, 19]. This implies that frequent vaginal examinations and fetal monitoring are painful for mothers. WHO guidelines emphasize the importance of informed consent and dignity in labor care, recommending that such procedures be performed only when necessary and with full maternal understanding [23].

Another critical challenge was the physical exhaustion caused by prolonged labor, food and sleep deprivation, and the emotional toll of uncertainty. Sleep deprivation in particular is known to negatively impact early postpartum recovery, maternal-infant bonding, and increase the risk of postpartum depression [24]. Ensuring adequate rest, nutrition, and psychological support is vital to improving postpartum well-being.

Finally, our study highlighted the lasting physical and psychological effects of IOL on mothers. Beyond the immediate postpartum period, mothers reported lingering stress, exhaustion, and in some cases, fear of future pregnancies. These findings are supported by research from Germany, where mothers expressed feelings of being forced into labor before their bodies were ready, affecting their recovery and perception of childbirth [19]. The dissonance between their expectations and actual experiences contributed to these negative outcomes. Counseling mothers about the postpartum implications of IOL may help them prepare and reduce long-term distress.

### Strength and limitation

This study is the first in our setting to focus specifically on mothers’ perspectives and lived experiences of childbirth following IOL. The inclusion of a pilot study was a notable strength, allowing for refinement of the research tools and ensuring clarity of interview questions. Additionally, we applied Guba’s trustworthiness criteria including credibility, dependability, confirmability, and transferability to ensure methodological rigor.

To minimize researcher bias, we also employed bracketing during data collection and analysis by consciously setting aside preconceived notions and personal experiences. However, we acknowledge that complete bracketing is inherently challenging in practice. While peer debriefing was used to enhance objectivity, the potential for subtle influence on interpretation cannot be entirely ruled out.

Nonetheless, other limitations remain. Some mothers may have withheld critical feedback, especially concerning healthcare providers due to concerns about how their responses might affect future care. This potential for social desirability bias underscores the importance of creating a safe, confidential, and non-judgmental interview environment in qualitative research.

## Conclusion

The findings of this study highlight that induced labor presents significant emotional, physical, and psychological challenges for mothers. Despite eventual happiness following successful deliveries, many participants experienced fear, pain, and stress that exacerbated by ward conditions and invasive procedures. However, support from healthcare providers and family members played a critical role in helping mothers endure the process. These insights underscore the need for more empathetic care practices, improved ward environments, and better preparatory counseling to enhance the experiences of mothers undergoing IOL.

## Recommendation

### For healthcare providers

Providers should ensure women are informed about the benefits, procedures, and possible complications of labor induction. Clear, respectful communication during antenatal care and before the induction is essential to build trust and support informed decision-making.

### Policy-level actions

Health institutions should implement standardized antenatal education protocols that ensure all women receive timely and consistent information about labor induction. Facilities should also introduce privacy protocols, during exams and labor. Improving staff-to-patient ratios through better staffing or scheduling will also support better communication and reduce delays in care.

### For hospitals

To improve the labor ward environment, efforts should focus on reducing overcrowding, maintaining cleanliness through regular cleaning, and improving privacy by separating laboring women from postpartum mothers and using sufficient screening materials.

### For researchers

Future studies should adopt mixed-method approaches to better capture both clinical outcomes and women’s lived experiences with labor induction.

## Data Availability

The datasets used in this study (transcripts, codes, and themes) are available from the corresponding author as per the guideline of the journal up on request.

## Appendix

**Table 2:** Socio-demographic characteristics of the participants, Wolaita Sodo university comprehensive specialized hospital, Southern Ethiopia, 2024 (*n* = 12)

Participant ID	Age	Education	Marital status	Monthly income (ETB)	Religion	Occupation	Residence	Number of children	Number of pregnancies
Code 01	25	Primary school	Married	6000	Protestant	House wife	Urban	02	02
Code 02	27	First degree	Married	10,000	Orthodox	Government employee	Urban	01	01
Code 03	27	Diploma	Married	8000	Protestant	Housewife	Urban	02	02
Code 04	27	First degree	married	9000	Protestant	Housewife	Urban	03	03
Code 05	30	First degree	Married	10,000	Protestant	Government employee	Urban	03	04
Code 06	30	Primary school	Married	3000	Protestant	Private employee	Urban	05	05
Code 07	26	First degree	Married	12,000	Protestant	Government employee	Urban	02	02
Code 08	23	First degree	Married	18,000	protestant	Government employee	Urban	01	01
Code 09	27	Secondary school	Married	7000	Orthodox	Housewife	Urban	01	01
Code 10	35	Diploma	Married	5000	Protestant	Government employee	Rural	03	03
Code 11	22	Secondary school	Married	4000	Orthodox	Private employee	Urban	01	02
Code 12	35	Secondary school	Married	9000	Protestant	Housewife	Urban	03	03

*ETB* Ethiopian birr

## Abbreviations

IOL: Induction of Labour
IUGR: Intrauterine Growth Restriction
GDM: Gestational Diabetes Mellitus
PROM: Premature Rupture of Membranes
PI: Principal Investigator
PPH: Postpartum Hemorrhage
SDG: Sustainable Development Goal
WHO: World Health Organization
WSUCSH: Wolaita Sodo University Comprehensive and Specialized Hospital

## References

[1] Lundh CØ, Øvrum AK, Dahl B. Women’s experiences with unexpected induction of labor: a qualitative study. Eur J Midwifery. 2023;7(7). 10.18332/ejm/161481. 10.18332/ejm/161481 PMC10037217 36970251

[2] World Health Organization. WHO labour care guide: user’s manual. 2020. https://www.who.int/publications/i/item/9789240017566.

[3] Debele TZ, Cherkos EA, Badi MB, Anteneh KT, et al. Factors and outcomes associated with the induction of labor in referral hospitals of Amhara regional state, Ethiopia: a multicenter study. BMC Pregnancy Childbirth. 2021:1–8. 10.1186/s12884-021-03709-5. 10.1186/s12884-021-03709-5 PMC8095340 33743637

[4] Zhu J, Xue L, Shen H, Zhang L, et al. Labor induction in China: a nationwide survey. BMC Pregnancy Childbirth. 2022;22(1):463. 10.1186/s12884-022-04760-6. 10.1186/s12884-022-04760-6 PMC9158355 35650545

[5] Henderson J, Redshaw M. Women’s experience of induction of labor: a mixed methods study. Acta Obstet Gynecol Scand. 2013;92(10):1159-67. 10.1111/aogs.12211. 10.1111/aogs.12211 23808325

[6] Gatward H, Simpson M, Woodhart L, Stainton MC. Women’s experiences of being induced for post-date pregnancy. Women Birth. 2010;23(1):3–9. 10.1016/j.wombi.2009.06.002. 10.1016/j.wombi.2009.06.002 19647506

[7] Waldenström U, Hildingsson I, Rubertsson C, Rådestad I. A negative birth experience: prevalence and risk factors in a national sample. Birth. 2004;31(1):17–27. 10.1111/j.0730-7659.2004.0270.x. 10.1111/j.0730-7659.2004.0270.x 15015989

[8] Shetty A, Burt R, Rice P, Templeton A. Women’s perceptions, expectations and satisfaction with induced labour a questionnaire-based study. Eur J Obstet Gynecol Reprod Biol. 2005; 123(1):56–61. 10.1016/j.ejogrb.2005.03.004. 10.1016/j.ejogrb.2005.03.004 15905017

[9] Maine D. Detours and shortcuts on the road to maternal mortality reduction. Lancet. 2007;370(9595):1380-2. 10.1016/S0140-6736(07)61580-3 17933653

[10] König-Bachmann M, Schwarz C, Zenzmaier C. Women’s experiences and perceptions of induction of labour: results from a German online-survey. Eur J Midwifery. 2017;1(2):1–8. 10.18332/ejm/76511.

[11] Gelling L. Qualitative research. Nurs Standard (2014+). 2015;29(30):43. 10.7748/ns.29.30.43.e9749 25804178

[12] World Health Organization. Maternal mortality measurement: guidance to improve national reporting. World Health Organization; 2022.

[13] Braun V, Clarke V. Using thematic analysis in psychology. Qual Res Psychol. 2006;3(2):77–101. 10.1191/1478088706qp063oa.

[14] Coates D, Donnolley N, Foureur M, Henry A. Women’s experiences of decision-making and attitudes in relation to induction of labour: a survey study. Women Birth. 2021;34(2):e170-7. 10.1016/j.wombi.2020.02.020. 10.1016/j.wombi.2020.02.020 32146087

[15] Coates R, Cupples G, Scamell A, McCourt C. Women’s experiences of induction of labour: qualitative systematic review and thematic synthesis. Midwifery. 2019;69:17–28. 10.1016/j.midw.2018.10.013. 10.1016/j.midw.2018.10.013 30390463

[16] Wigert H, Nilsson C, Dencker A, Begley C, et al. Women’s experiences of fear of childbirth: a metasynthesis of qualitative studies. Int J Qual Stud Health Wellbeing. 2020;15(1):1704484. 10.1080/17482631.2019.1704484. 10.1080/17482631.2019.1704484 PMC6968519 31858891

[17] Murtagh M, Folan M. Women’s experiences of induction of labour for post-date pregnancy. Br J Midwifery. 2014;22(2):105−10. 10.12968/bjom.2014.22.2.105.

[18] Gauci Y, Spiteri G. Mothers’ experiences of induction of labour. Malta J Health Sci. 2022;9(26):18–26. 10.14614/LABOUR/9/22.

[19] Atobrah-Apraku K, Newman GT, Opuni-Frimpong Y, Seffah JD, Adu-Bonsaffoh K. Lived experiences of women during induction of labour at a tertiary hospital in Ghana: a qualitative study. PLOS Glob Public Health. 2024;4(2):e0002290. 10.1371/journal.pgph.0002290 PMC10868737 38359028

[20] Bohren MA, Hofmeyr GJ, Sakala C, Fukuzawa RK, Cuthbert A. Continuous support for women during childbirth. Cochrane Database Syst Rev. 2017; 7(7):CD003766. 10.1002/14651858.CD003766.pub6. PMID: 28681500; PMCID: PMC6483123. 10.1002/14651858.CD003766.pub6 PMC6483123 28681500

[21] Moyer CA, Adongo PB, Aborigo RA, Hodgson A, Engmann CM. ‘They treat you like you are not a human being’: maltreatment during labour and delivery in rural northern Ghana. Midwifery. 2014; 30(2):262-8. 10.1016/j.midw.2013.05.006. Epub 2013 Jun 19. PMID: 23790959. 10.1016/j.midw.2013.05.006 23790959

[22] Lou S, Hvidman L, Uldbjerg N, Neumann L, et al. Women‘s experiences of postterm induction of labor: a systematic review of qualitative studies. Birth. 2019;46(3):400−10. 10.1111/birt.12412. Epub 2018 Dec 18. 10.1111/birt.12412 30561053

[23] WHO. WHO recommendations: intrapartum care for a positive childbirth experience. 2018. 30070803

[24] Hunter LP, Rychnovsky JD, Yount SM. A selective review of maternal sleep characteristics in the postpartum period. J Obstet Gynecol Neonatal Nurs. 2009;38(1):60−8. 10.1111/j.1552-6909.2008.00309.x. PMID: 19208049. 10.1111/j.1552-6909.2008.00309.x 19208049

